# The H^+^-Translocating Inorganic Pyrophosphatase From *Arabidopsis thaliana* Is More Sensitive to Sodium Than Its Na^+^-Translocating Counterpart From *Methanosarcina mazei*

**DOI:** 10.3389/fpls.2020.01240

**Published:** 2020-08-12

**Authors:** José R. Pérez-Castiñeira, Aurelio Serrano

**Affiliations:** Instituto de Bioquímica Vegetal y Fotosíntesis, Universidad de Sevilla-CSIC, Sevilla, Spain

**Keywords:** *Arabidopsis thaliana*, heterologous expression, membrane-bound ion (H^+^- or Na^+^)-pumping inorganic pyrophosphatases, *Methanosarcina mazei*, *Saccharomyces cerevisiae*, salt stress, soluble inorganic pyrophosphatase

## Abstract

Overexpression of membrane-bound K^+^-dependent H^+^-translocating inorganic pyrophosphatases (H^+^-PPases) from higher plants has been widely used to alleviate the sensitivity toward NaCl in these organisms, a strategy that had been previously tested in *Saccharomyces cerevisiae*. On the other hand, H^+^-PPases have been reported to functionally complement the yeast cytosolic soluble pyrophosphatase (IPP1). Here, the efficiency of the K^+^-dependent Na^+^-PPase from the archaeon *Methanosarcina mazei* (MVP) to functionally complement IPP1 has been compared to that of its H^+^-pumping counterpart from *Arabidopsis thaliana* (AVP1). Both membrane-bound integral PPases (mPPases) supported yeast growth equally well under normal conditions, however, cells expressing MVP grew significantly better than those expressing AVP1 under salt stress. The subcellular distribution of the heterologously-expressed mPPases was crucial in order to observe the phenotypes associated with the complementation. *In vitro* studies showed that the PPase activity of MVP was less sensitive to Na^+^ than that of AVP1. Consistently, when yeast cells expressing MVP were grown in the presence of NaCl only a marginal increase in their internal PPi levels was observed with respect to control cells. By contrast, yeast cells that expressed AVP1 had significantly higher levels of this metabolite under the same conditions. The H^+^-pumping activity of AVP1 was also markedly inhibited by Na^+^. Our results suggest that mPPases primarily act by hydrolysing the PPi generated in the cytosol when expressed in yeast, and that AVP1 is more susceptible to Na^+^ inhibition than MVP both *in vivo* and *in vitro*. Based on this experimental evidence, we propose Na^+^-PPases as biotechnological tools to generate salt-tolerant plants.

## Introduction

The membrane-bound K^+^-dependent H^+^-translocating inorganic pyrophosphatase (H^+^-PPase), encoded by *AVP1*, has been reported to be the protein responsible for removing most of the inorganic pyrophosphate (PPi) generated in the cytosol of *Arabidopsis thaliana* cells (Ferjani et al., 2011; Kriegel et al., 2015; Segami et al., 2018b). The removal of PPi, a by-product of many biosynthetic reactions, is essential in all living cells because its accumulation causes the collapse of anabolism, eventually leading to cell death (Lahti, 1983; Serrano-Bueno et al., 2013). AVP1 couples the energy released by the hydrolysis of PPi to the translocation of protons into the vacuole, thus acting as a second proton pump in the plant tonoplast along with the V-type H^+^-ATPase (Sarafian et al., 1992; Graus et al., 2018). By contrast, in the cytosol of *Saccharomyces cerevisiae* cells only one protein seems to have the capacity to hydrolyse PPi: the extremely efficient soluble inorganic pyrophosphatase (sPPase), encoded by *IPP1* (Kolakowski et al., 1988).

In a seminal paper published in 2001, Gaxiola and co-workers reported that overexpression of AVP1 alleviated the sensitivity of *A. thaliana* to NaCl (Gaxiola et al., 2001). Thereafter, several groups obtained other plants with higher tolerance to salinity and other stresses based on this approach (Park et al., 2005; Gao et al., 2006; Li et al., 2008; Lv et al., 2008; Pasapula et al., 2011; Gaxiola et al., 2012; Schilling et al., 2014; Yang et al., 2015; Lv et al., 2016; Ahire et al., 2018). This strategy had been originally tested in *S. cerevisiae* (Gaxiola et al., 1999), thus demonstrating the suitability of this yeast to study and test solutions to salt stress in plants.

Although AVP1 and its orthologs in higher plants have been used as tools to develop salt-tolerant plants, membrane-bound PPases with different biochemical properties have been characterized from other organisms. A K^+^-independent H^+^-PPase was identified in 1966 in the photosynthetic alphaproteobacterium *Rhodospirillum rubrum* (RVP) (Baltscheffsky and Baltscheffsky, 1995). The corresponding gene was cloned and sequenced in the 1990s (Baltscheffsky et al., 1998) and expressed both in *E. coli* (Schultz and Baltscheffsky, 2004) and *S. cerevisiae* (Drake et al., 2010). Two ortologs of RVP have been identified in *A. thaliana* (AVP2 and AVP3) and reported to be located in the Golgi membranes, however, these proteins seem to have much lower expression levels than AVP1 and their physiological importance remains to be established (Drozdowicz et al., 2000; Ferjani et al., 2011). An important breakthrough in the field of PPases was the discovery of K^+^-dependent Na^+^-translocating mPPases (Na^+^-PPases) in eubacteria and archaea (Malinen et al., 2007; Baykov et al., 2013). Nowadays, genome projects of a wide variety of organisms as well as metagenomics and biochemical studies are significantly broadening the Na^+^-PPases field (Luoto et al., 2015; Serrano A., unpublished).

Our group has previously reported the generation of several *S. cerevisiae* mutant strains whose *IPP1* genes are under the control of the glucose-repressible promoter of the *GAL1* gene. These strains cannot grow on glucose, thus demonstrating the essentiality of the sPPase encoded by *IPP1* (Serrano-Bueno et al., 2013), however, the capacity to grow on this carbon source is recovered when they are transformed with plasmids bearing genes coding for H^+^-PPases under the control of constitutive promoters (Pérez-Castiñeira et al., 2002; Drake et al., 2010; Pérez-Castiñeira et al., 2011; Hernández et al., 2015). This system avoids the competition for the substrate between the heterologous H^+^-PPase and the yeast cytosolic sPPase *in vivo*, a situation that resembles that of a plant cell cytosol.

In this communication, the capacity of two different mPPases to complement IPP1 under salt stress has been studied. In order to accomplish this task, several yeast mutants with different sensitivities to NaCl and conditional expression of IPP1 were generated. These mutants were transformed with plasmids bearing genes coding for AVP1 or the K^+^-dependent Na^+^-PPase from the salt-tolerant archaeon *Methanosarcina mazei* (MVP) (Malinen et al., 2007). MVP was more efficient than AVP1 at supporting growth of the different yeast mutants in the presence of NaCl. The addition of N-terminal signal peptides that modify the subcellular distribution of the mPPases (Drake et al., 2010) was essential for efficient complementation of IPP1. *In vitro* assays showed that AVP1 is significantly more sensitive to Na^+^ than MVP.

Our results suggest that mPPases may be direct targets of Na^+^ *in vivo*, a situation that might lead to a toxic accumulation of PPi in the cytosol of plant cells subjected to high concentrations of salt. Based on this evidence, we propose the heterologous expression of chimeric versions of Na^+^-PPases with appropriate N-terminal signal peptides as a more efficient way to improve salt-tolerance in plants than the current approach, based on the use of untargeted H^+^-PPases. In connection with this, *in silico* analyses of currently available genomes suggest that Na^+^-PPases, originally described in bacteria and archaea (Malinen et al., 2007; Luoto et al., 2011; Luoto et al., 2015), are present in other organisms, including some of the photosynthetic lineage like certain marine microalgal groups (Prasinophyceae and diatoms). Our findings suggest new biotechnological applications for microalgae in order to improve plants of agronomical interest.

## Materials and Methods

### Yeast Strains

The strains used in this work are shown in **Table 1**. YPC3 mutant strain was generated from *S. cerevisiae* haploid strain W303-1A (MATa, *ade2-1 can1-100 his3-11,15 leu2-3,112 trp1-1*, *ura3-1*) by the single-step transplacement procedure as previously described (Drake et al., 2010). This mutant has *IPP1*, the gene that codes for the cytosolic soluble inorganic PPase, under the control of the yeast galactokinase gene (*GAL1*) promoter (Drake et al., 2010). YPC5 and YPC6 mutant strains were obtained from *S. cerevisiae* strains G19 and B31, respectively, by following the same strategy except that the *TRP7* cassette was used as a selection marker instead of *HIS3.* Mutant G19 had been derived from strain W303-1B by disrupting its *ENA1-4* genes with the yeast *HIS3* cassette (Quintero et al., 1996), whereas B31 was obtained from G19 by disrupting *NHA1* with the *LEU2* cassette (Bañuelos et al., 1998). Both mutants were generously provided by Professor Alonso Rodríguez Navarro (Universidad Politécnica de Madrid, Spain).

**Table 1:** Yeast strains used in this work.

Name	Relevant genotype	Reference
W303-1A	*MATa, ade2-1 can1-100 his3-11,15 leu2-3,112 trp1-1 ura3-1*	(Thomas and Rothstein, 1989)
G19	MATα*, ade2-1 can1-100 his3-11,15 leu2-3,112 trp1-1 ura3-1 ena1Δ::HIS3::ena4Δ*	(Quintero et al., 1996)
B31	*MATα, ade2-1 can1-100 his3-11,15 leu2-3,112 trp1-1 ura3-1 ena1Δ::HIS3::ena4Δ nha1Δ::LEU2*	(Bañuelos et al., 1998)
YPC3	W303-1A *ipp1_UAS_-ipp1_TATA_::HIS3-GAL1_UAS_-GAL1_TATA_-IPP1*	(Drake et al., 2010)
YPC5	G19 *ipp1_UAS_-ipp1_TATA_::TRP1-GAL1_UAS_-GAL1_TATA_-IPP1*	This study
YPC6	B31 *ipp1_UAS_-ipp1_TATA_::TRP1-GAL1_UAS_-GAL1_TATA_-IPP1*	This study

### Plasmids Construction and Yeast Transformation

YPC3, YPC5, and YPC6 cells were transformed with the plasmids shown in **Table 2** as previously described (Schiestl and Gietz, 1989). All plasmids are 2 micron-based derivatives of the *URA3*-containing *E. coli/S. cerevisiae* shuttle plasmid pRS699, that bears the yeast *PMA1* promoter for constitutive expression of inserts (Serrano and Villalba, 1995). The latter were made by in-frame fusion of the N-terminal signal peptides of *S. cerevisiae* SUC2 invertase (Inv) or the K^+^-dependent H^+^-PPase from *Trypanosoma cruzi* (Tc), the sequence coding for the yeast-enhanced green fluorescent protein (yEGFP) and the coding sequence of the K^+^-dependent H^+^-PPase from *A. thaliana* (AVP1) (Drake et al., 2010). An identical approach was followed with the coding sequence of the K^+^-dependent Na^+^-PPase from *M. mazei* (MVP) (Malinen et al., 2007), generously provided by Professor Reijo Lahti, University of Turku (Finland).

**Table 2:** Plasmids used for yeast transformation.

Plasmid	Insertion	Reference
pIPP1	Sequence coding for the cytosolic soluble PPase from *S. cerevisiae*	(Pérez-Castiñeira et al., 2002)
pInvGFPAVP1	Sequence coding for the putative N-terminal signal peptide of Suc2p followed by those of yEGFP and the H^+^-PPase from *A. thaliana* (AVP1)	(Drake et al., 2010)
pInvGFPMVP	Sequence coding for the putative N-terminal signal peptide of Suc2p followed by those of yEGFP and the Na^+^-PPase from *M. mazei* (MVP)	This study
pTcGFPAVP1	Sequence coding for the putative N-terminal signal peptide of the K^+^-dependent H^+^-PPase from *Trypanosoma cruzi* followed by those of yEGFP and AVP1	(Drake et al., 2010)
pTcGFPMVP	Sequence coding for the putative N-terminal signal peptide of the K^+^-dependent H^+^-PPase from *T. cruzi* followed by those of yEGFP and MVP	This study

Original and chimeric nucleotide sequences described in the table were inserted between yeast PMA1 promoter and terminator of the URA3-containing plasmid pRS699 (Serrano and Villalba, 1995).

For the transformation procedure, YPC3, YPC5 and YPC6 cells were initially grown at 30°C in YPGal liquid medium [1% (w/v) yeast extract, 2% (w/v) peptone, 2% (w/v) galactose], while transformants were selected on 2% agar plates made in galactose-containing synthetic medium [0,17% (w/v) yeast nitrogen base without amino acids and ammonium sulphate, 2% (w/v) galactose, 50 mM TRIS-HCl pH 6, and a mixture of nucleotides and amino acids described in (Treco and Lundblad, 2001)], devoid of histidine and uracil (YPC3); histidine, tryptophan, and uracil (YPC5); or histidine, tryptophan, leucine, and uracil (YPC6). Colonies appeared after incubating the plates for 3-4 days at 30°C.

### Phenotype Complementation Tests

Complementation studies were performed by inoculating 2 ml of galactose-containing selective medium with transformed cells from the plates and growing overnight at 30°C on an orbital shaker (150 r.p.m.). The following day, 10 μl of these cultures were used to inoculate 2 ml of YPD medium [1% (w/v) yeast extract, 2% (w/v) peptone, 2% (w/v) glucose] (1:200 dilution) and allowed to grow overnight as described above. This treatment is necessary to bring down the PPase activity associated with the chromosome-encoded IPP1, controlled by the glucose-repressible *GAL1* promoter (Pérez-Castiñeira et al., 2002). After overnight growth on glucose, ten-fold serial dilutions of the cultures were made in sterile water and 5 μl drops of each dilution were spotted onto YPD agar plates containing different NaCl concentrations and 50 mM TRIS-MES adjusted to pH 6. Plates were incubated at 30°C for several days.

### Isolation of Yeast Microsomal Membranes

Preparations of membrane fractions were obtained from YPC6 cells transformed with the different plasmids by a modification of a method previously described (Serrano and Villalba, 1995): yeast colonies were collected from a plate and grown up to stationary phase in galactose-containing selective liquid medium; then, 400 ml of YPD were inoculated with 2 ml of stationary cultures. After overnight growth, cells were collected by centrifugation at 700 x *g* for 10 min, washed thoroughly with water, resuspended in 5 ml of ice-cold buffer A (25 mM Tris-HCl, pH 8, 10% glycerol, 4 mM β-mercaptoethanol, 2 mM DTT, 1 mM benzamidine, 2 mM ϵ-aminocaproic acid, 1 mM PMSF), and homogenized by vigorous vortexing for five one-min bursts in the presence of glass beads (0.5 mm Ø) with one-min intervals on ice. From this point on, the whole procedure was carried out at 0°C (except centrifugations, that were performed at 4°C). The homogenate was diluted up to 25 ml with buffer B (10 mM Tris-HCl, pH 7.6, 10% (w/v) glycerol, 2 mM DTT) and centrifuged for 10 min at 700 x *g* to remove beads and debris. The resulting supernatant was centrifuged for 20 min at 20,000 x *g* and the pellet [the crude mitochondrial fraction, that includes mitochondria and plasma membrane vesicles among others (Serrano and Villalba, 1995)] was discarded for PPi hydrolysis assays due to the potential interference of the mitochondrial PPase (Lundin et al., 1991). The supernatant of this step was centrifuged for 30 min at 120,000 x *g*. The pellet thus obtained, the microsomal fraction, was taken up, homogenized in stripping buffer [60 mM Tris/HCl, pH 8, 12% (w/v) glycerol, 0.72 M KCl, and 1.2 mM CaCl_2_ (Lenoir et al., 2002)] and centrifuged for 20 min at 120,000 x *g*. Finally, the stripped microsomal fraction was washed with buffer B and centrifuged (20 min, 120,000 x *g*). The final pellet was resuspended and homogenized in 0.5–1 ml of buffer B. Microsomes were directly used for activity assays or divided into aliquots and kept at -80°C until use.

### Pyrophosphatase Activity and ACMA Quenching Assays, Western Blot, and Total Protein Estimation

Pyrophosphatase (PPase) activity was assayed at 30°C by incubation of protein preparations with Mg_2_PPi at pH 7.2 with the addition of KCl, NaCl and/or NaF when indicated, and colorimetrically measuring the released ortophosphate as previously described (Rathbun and Betlach, 1969). Mg_2_PPi was obtained by adding fixed volumes of stock solutions of appropriate concentrations of MgCl_2_ and Na_4_PPi to the assay mixes. Reactions were started by the addition of the latter and stopped with a mixture of trichloroacetic acid, sodium acetate, acetic acid, and formaldehyde (final concentrations: 4% (w/v), 0,5 M, 0.5 M, and 1% (w/v), respectively) (Schultz and Baltscheffsky, 2004). Control yeast microsomal membranes devoid of PPase activity and containing the same amount of total protein were used as blanks in every assay. ACMA quenching assays and *Western blot*s were performed as described elsewhere (Perez-Castiñeira and Apps, 1990; Valverde et al., 1997). A polyclonal antibody raised against the purified Na^+^-PPase from the marine bacterium *Thermotoga maritima* (TVP) (López-Marqués et al., 2005) and a commercial polyclonal rabbit antibody against yeast IPP1 protein (AP21326F_N, OriGene EU, Germany) were used for immunodetection of mPPases and sPPases, respectively, after SDS-PAGE and semi-dry electroblotting transfer. Antibody against TVP was affinity-purified as previously described (Pringle et al., 1991). Protein concentration was estimated using a Coomassie-blue dye binding-based assay from Bio-Rad (München, Germany) following manufacturer’s instructions and using ovalbumin as a standard.

### Analysis of Enzyme Kinetics

Kinetic data obtained for AVP1 and MVP were fitted to the Hill equation and to a mathematical expression based on the concerted MWC allosteric model (see Results) by weighted non-linear least squares (nls) regression using RStudio (version 1.1.456 – ^©^ 2009–2018 RStudio, Inc.), downloaded from https://cran.cnr.berkeley.edu. This version of RStudio is an integrated development environment (IDE) for the programming language R, version 3.5.1 (2018-07-02), “Feather Spray” (^©^ 2018 The R Foundation for Statistical Computing), and was run under Ubuntu 18.04.1 LTS (“Bionic Beaver”) and Debian GNU/Linux 10 (“buster”). Averages of three to five independent experiments were used for regression calculations and each experimental data point was weighted as 1/variance.

### Determination of Internal PPi Levels in Yeast Cells

100 ml cultures of YPC6 cells transformed with the plasmids shown in **Table 2** and treated to minimize the chromosome-encoded IPP1 (see above) were grown in glucose-containing medium (YPD adjusted to pH 6) with agitation in the presence or absence of 100 mM NaCl. The initial optical densities at 660 nm (OD_660_) of the cultures where individually adjusted so that all of them had an OD_660_ of around 0.5 (roughly 10^9^ cells) after 8–9 h. Then, cells were collected by centrifugation, washed with ice-cold deionised water and broken as described for isolation of yeast membranes, except that a 4% (v/v) perchloric acid aqueous solution was used instead of buffer A. Beads, debris, and denatured proteins were removed by centrifugation (20 min, 20,000 × g*, 4 °C*) and PPi concentrations were determined colorimetrically in the supernatants after neutralization with KOH, as previously described (Heinonen et al., 1981).

### Molecular Phylogenetic Analysis of mPPases

The amino acid sequences of selected H^+^- and Na^+^-translocating mPPases orthologs of bacteria, archaea, parasitic protists, microalgae and plants were aligned with the CLUSTAL X software tool (Larkin et al., 2007). Phylogenetic trees were constructed with the evolutionary distances (neighbor joining) and maximum parsimony methods using the SeaView v5.2 software (Gouy et al., 2010).

## Results

### MVP Functionally Complements Yeast IPP1 More Efficiently Than AVP1 Under Salt Stress

Three yeast strains with increasing sensitivities to NaCl were generated (**Table 1**) and transformed with *E. coli/S. cerevisiae* shuttle plasmids bearing the genes coding for chimeric versions of AVP1 and MVP under the control of the yeast *PMA*1 promoter (**Table 2**). Drop tests to check for growth of transformants onto YPD plates at pH 6 containing different concentrations of NaCl were carried out.

We have previously shown that addition of certain sequences in the N-termini of the coding sequences of some mPPases altered the subcellular distribution of the resulting chimeric peptide and improved their expression levels. Among these sequences were those encoding the N-terminal signal peptides from the *S. cerevisiae* invertase SUC2 or the K^+^-dependent H^+^-PPase from *Trypanosoma cruzi* (TcVP), which targeted the chimeric mPPases mainly to the plasma membrane or the internal membrane systems, respectively. Fusion with the yEGFP sequence further enhanced mPPase expression levels, especially in the case of AVP1 (Drake et al., 2010; Pérez-Castiñeira et al., 2011). This protein engineering strategy was applied to the coding sequence of the K^+^-dependent Na^+^-PPase from *M. mazei* with similar results (**Supplementary Figure S1**).

**Figure 1:**
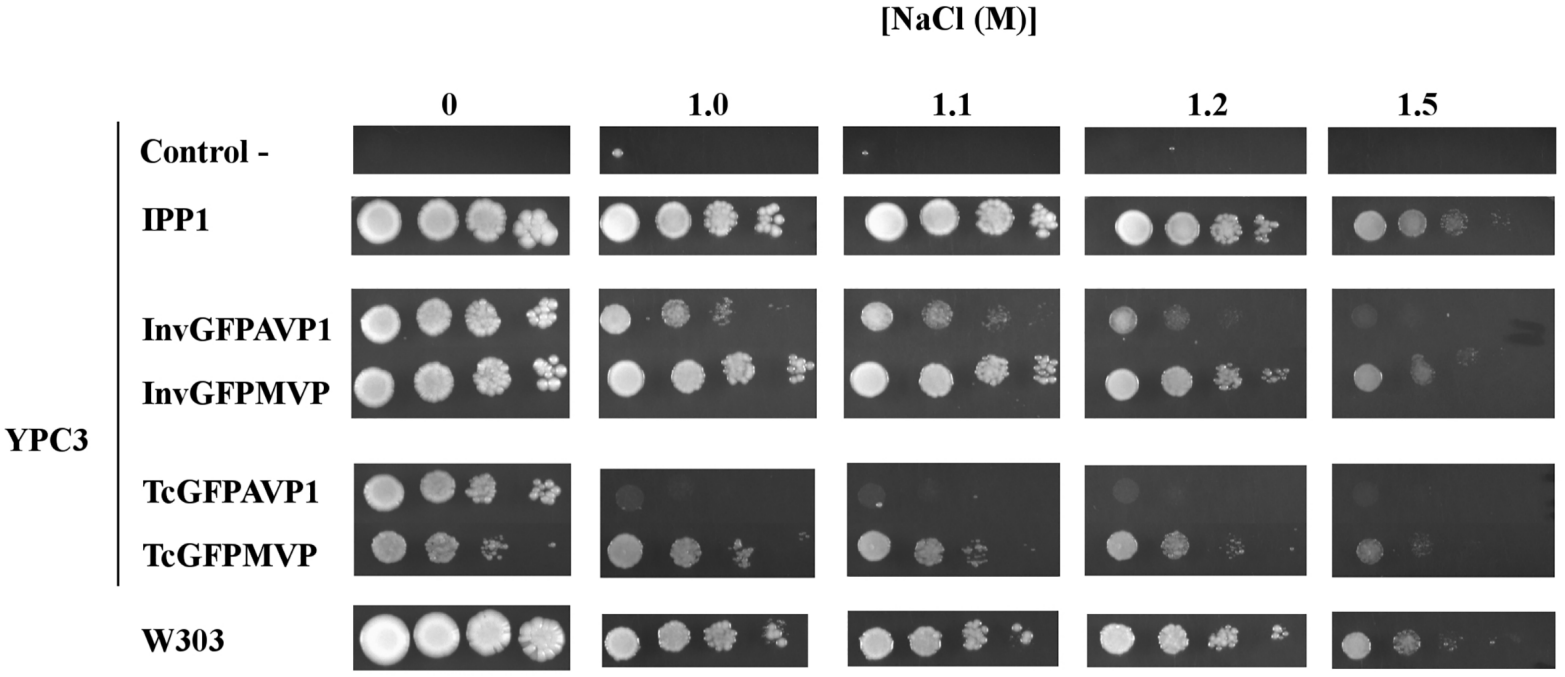
Drop tests for yeast mutant YPC3 cells and parental strain W303 grown in the presence different concentrations of NaCl. Cells were transformed with plasmids shown in and grown as described in the main text. Serial dilutions of the cultures were made in sterile water and spotted on to YPD plates adjusted to pH 6 and containing the indicated concentrations of NaCl. Growth was recorded after 4 days at 30 °C. Negative controls are YPC3 cells transformed with empty plasmid.

Initial studies of growth in the presence of NaCl were done with YPC3, a mutant derived from the strain W303-1A, which is not especially sensitive to Na^+^ and is able to grow in the presence of NaCl concentrations in the molar range (Rios et al., 1997). **Figure 1** shows drop tests comparing growth obtained with YPC3 cells transformed with plasmids bearing different PPase genes and with the parental strain W303-1A. Cells expressing MVP grew significantly better than those expressing AVP1 in the presence of NaCl, however, no difference was observed in the absence of salt. Moreover, the chimera with the N-terminal signal peptide of yeast invertase SUC2 (InvGFPMVP) performed better than that with the N-terminal signal peptide of TcVP (TcGFPMVP). InvGFPAVP1 also supported growth more efficiently than TcGFPAVP1, albeit at lower NaCl concentrations than the MVP chimeras. None of the cells expressing mPPases grew better than those expressing their soluble counterpart from yeast (IPP1) under any of the conditions tested.

**Figure 2:**
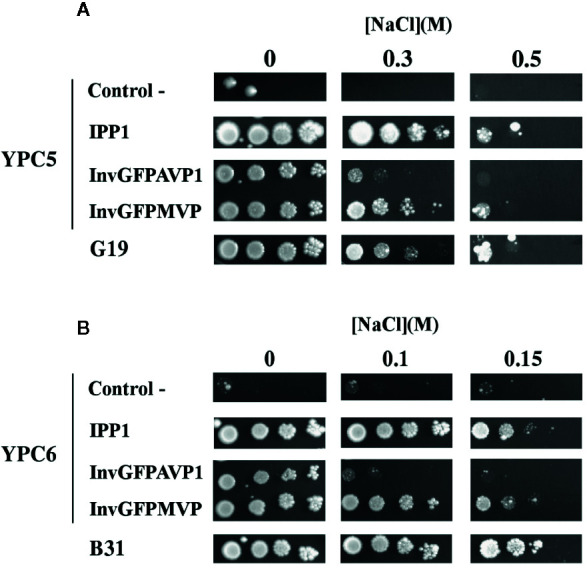
Drop tests for sodium-sensitive yeast strains YPC5 **(A)**, YPC6 **(B)**, and their respective parental strains G19 and B31. Cells were transformed with plasmids pIPP1, pInvGFPAVP1 and pInvGFPMVP as described in the main text. The rest of conditions as described in . Negative controls are YPC5 **(A)** and YPC6 **(B)** cells transformed with empty plasmid.

The use of YPC3 mutant and its parental strain forced the addition of very high concentrations of NaCl to observe the effects exerted by this salt (above 1 M). At such high concentrations, NaCl poses two types of stress to yeast cells, namely, osmotic stress and sodium toxicity. Although in glucose-containing medium the latter contributes more to growth inhibition than the former (Rios et al., 1997), we were interested in the specific effects of Na^+^. It was then decided to use yeast strains that were sensitive to lower NaCl concentrations in order to minimize osmotic stress, therefore, we generated mutant strains YPC5 and YPC6. These strains have conditional expression of their respective *IPP1* genes in genetic backgrounds associated with higher sensitivities to NaCl (knock-outs of genes encoding major sodium pumps and/or transporters; **Table 1**). YPC5 and YPC6 cells were transformed with the plasmids described in **Table 2** and the results obtained are shown in **Figures 2A, B**, respectively. An identical pattern to that obtained in the case of transformed YPC3 cells was observed, the only difference being the concentrations of NaCl required to inhibit growth. YPC5 cells stopped growing at around 0.5 M NaCl, whereas 150 mM NaCl was sufficient to obtain a similar effect in YPC6 cells. As far as mPPases are concerned, the chimera InvGFPMVP supported growth more efficiently than InvGFPAVP1, and both performed better than TcGFPMVP and TcGFPAVP1, respectively (not shown for clarity), as in the case of YPC3. YPC5 and YPC6 cells expressing IPP1 always performed better than those expressing chimeric mPPases. YPC6 cells transformed with plasmids pInvGFPMVP and pInvGFPAVP1 were selected for subsequent studies.

The results obtained with YPC3, YPC5 and YPC6 showed that the capacity of AVP1 to complement IPP1 was severely hampered by the presence of NaCl in the medium, whereas that of MVP was less sensitive to this circumstance. The fact that identical results were obtained with NaCl concentrations ranging from 1.5 M down to 150 mM suggests that the effects exerted by NaCl are actually due to Na^+^ toxicity rather than to osmotic stress. This idea was supported by drop tests performed with transformed YPC5 and YPC6 cells in YPD plates supplemented with 1 M and 0.3 M sorbitol, that showed no difference with respect to those performed in YPD alone (not shown). Moreover, the addition of KCl instead of NaCl in the culture medium did not inhibit growth in any case (not shown), demonstrating that the phenotypes observed in the presence of NaCl were due to the toxic effects of Na^+^.

### Membrane-Associated PPase Activity and Expression Levels of the Heterologously-Expressed mPPases Do Not Change When Yeast Cells Are Subjected to Salt Stress

**Figure 3:**
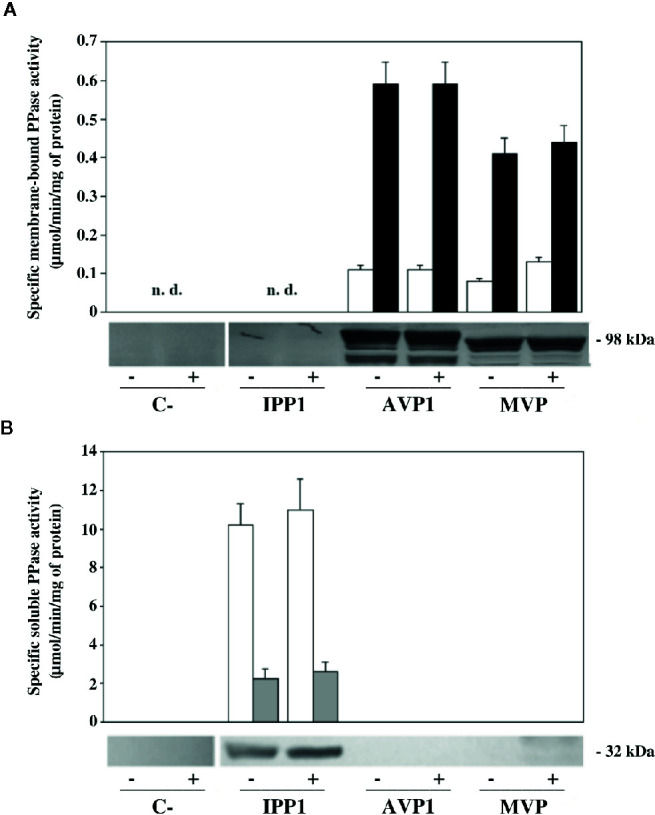
Levels of PPi hydrolysis activity and immunodetection of mPPases in microsomal membranes **(A)** and sPPases in soluble extracts **(B)** of YPC6 mutant cells transformed with plasmids shown in **Table 2**. Cells were cultured in liquid YPD up to an OD_660nm_ of 0.3, as described in main text, then, each culture was divided into two aliquots, one of which was subjected to a treatment with 100 mM NaCl by adding the appropriate volume of a 5 M stock solution of NaCl, whereas the same volume of deionized water was added to the other aliquot. All cultures were grown up to an OD_660nm_ of 0.8-1, cells were collected and microsomal membranes and soluble crude extracts were obtained as described in Materials and Methods. An affinity purified polyclonal antibody against *Thermotoga maritima* mPPase **(A)** and a commercial antibody against IPP1 **(B)** were used for immunodetection. 70 μg of total protein were loaded per lane in all cases. Positive (+) signs denote samples obtained from cells subjected to treatment with 100 mM NaCl. Negative (-) signs denote samples obtained from control cultures with no added salt. “n. d.”: not detected.

In order to check whether the phenotypes observed could be due to differences in protein expression and/or PPase activity, different studies were done with microsomal membrane preparations and soluble extracts obtained from transformed YPC6 cells. The upper panel of **Figure 3A** shows fluoride-insensitive PPi hydrolysis activity associated with microsomal membranes. As expected, PPase activity could only be detected in preparations obtained from cells transformed with plasmids bearing genes coding for mPPases. These activities increased 6 to 7-fold in the presence of 100 mM KCl *in vitro*, a feature of both AVP1 and MVP (Kim et al., 1994; Malinen et al., 2007). The presence of NaCl during growth did not alter *in vitro* activity levels.

No membrane-bound PPase activity was detected in YPC6 control cells (transformed with empty plasmid pRS699), whereas in membrane preparations of cells transformed with plasmid pIPP1 some residual activity was detected in the absence of NaF (not shown). This indicates that some molecules of the yeast cytosolic sPPase remain attached to these membranes even after treatment with stripping buffer, as sensitivity to fluoride is a characteristic of this enzyme (see below).

Immunodetection carried out with microsomal membranes using an affinity-purified polyclonal antibody against the Na^+^-PPase from *T. maritima* (TVP) confirmed the results obtained with PPase assays, that is, salt stress does not significantly alter expression levels and/or protein processing patterns in any case (**Figure 3A**, lower panel). The latter are due to partial degradation of the heterologously-expressed mPPases by yeast vacuolar proteases *in vivo* and it is a characteristic of our expression system (Pérez-Castiñeira et al., 2011).

A parallel set of experiments were carried out with soluble protein extracts of YPC6 cells transformed with the same plasmids as above. Fluoride-sensitive sPPase activity could be measured only in extracts from cells transformed with plasmid pIPP1, consistently, a commercial antibody against IPP1 recognized a band of the expected size (ca. 37 kDa) in this sample (**Figure 3B**). Similar results were obtained with cells of the parental strain B31, albeit with lower levels of activity and IPP1 polypeptide (not shown). Growing cells in the presence of NaCl did not significantly alter IPP1 expression levels.

### Membrane-Bound PPases Exhibit Different Sensitivities to NaCl *In Vitro*

**Figure 4:**
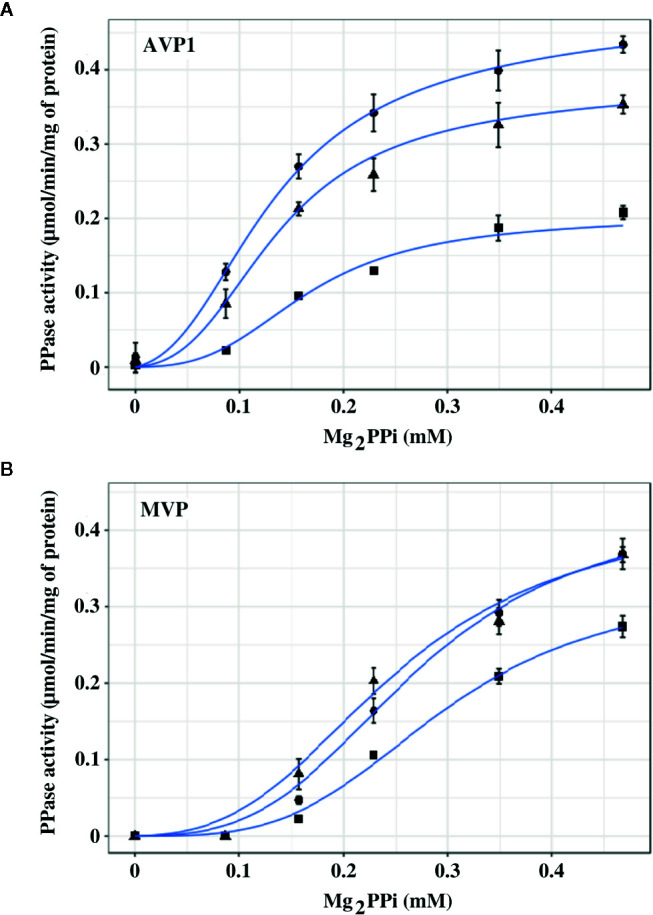
Graphical representation of PPase activity versus substrate (Mg_2_PPi) concentration for AVP1 **(A)** and MVP **(B)** in the presence of 0 mM (●), 50 mM (▲), and 100 mM (■) NaCl. Data points correspond to the averages ± standard errors (SE) of 4 independent experiments. The substrate, Mg_2_PPi, was obtained by mixing stock solutions of Na_4_PPi and MgCl_2_, as described in Materials and Methods, consequently, basal concentrations of Na^+^ four times higher than those of PPi are present in the assays. Mg_2_PPi concentrations were estimated considering the dissociation constants for individual PPi complexes with H^+^, Na^+^, K^+^, and Mg^2+^, as previously described (Baykov et al., 1993). Graph lines were calculated with Equation 1 using RStudio, as described in Materials and Methods.

The effect exerted by Na^+^ on the PPase activities of AVP1 and MVP *in vitro* was studied by performing assays in the presence of increasing concentrations (0, 50, and 100 mM) of added NaCl. It must be borne in mind that PPi is added as Na_4_PPi (see Materials and Methods), therefore, basal concentrations of Na^+^ four times higher than those of PPi are always present in the assays. Both AVP1 and MVP depend on K^+^ for full activity (Kim et al., 1994; Malinen et al., 2007), therefore, 100 mM KCl was always present in the assay mix to provide the concentration of K^+^ usually maintained in most plant cells (Nieves-Cordones et al., 2016). Mg_2_PPi was used as substrate, as previously reported for diverse mPPases (Leigh et al., 1992; Gordon-Weeks et al., 1996; Tsai et al., 2014). Results obtained are shown in **Figure 4**. AVP1 seemed to be quite sensitive to NaCl, showing a decrease in V_m_ of 20% and 50% in the presence of 50 and 100 mM NaCl, respectively; by contrast, MVP showed virtually no inhibition at 50 mM NaCl and only a 20% decrease in this parameter when the salt concentration was raised up to 100 mM.

The experimental data were fitted with the empirical Hill equation (Hill, 1910) by weighted non-linear least squares regression using RStudio, yielding values of the Hill coefficient *n_H_* of 2–3 for AVP1 and 3.5–4.5 for MVP. This indicates a significant degree of positive cooperativity for both proteins, MVP showing a higher deviation from the Michaelis-Menten behaviour (that is, higher sigmoidicity) than AVP1. The V_m_ value predicted by the Hill equation for MVP decreased somewhat with increasing concentrations of NaCl, however, neither *n_H_* nor the K_0.5_ values changed significantly (**Table 3A**).

**Table 3:** Kinetic values obtained by fitting the Hill equation (A) and Equation 1 (B) to the experimental data shown in **Figure 4**.

**(A)**			
**AVP1**	**[NaCl] (mM)**		
**Parameter**	**0**	**50**	**100**
V_m_ (U/mg prot.)	0.47 ± 0.01	0.37 ± 0.01	0.23 ± 0.01
K _0.5_ (mM)	0.14 ± 0.08	0.14 ± 0.11	0.19 ± 0.17
n_H_	2.06 ± 0.14	2.45 ± 0.22	2.63 ± 0.34
**MVP**	**[NaCl] (mM)**		
**Parameter**	**0**	**50**	**100**
V_m_ (U/mg prot.)	0.41 ± 0.04	0.35 ± 0.04	0.33 ± 0.01
K _0.5_ (mM)	0.27 ± 0.27	0.20 ± 0.27	0.30 ± 0.25
n_H_	3.74 ± 0.60	4.27 ± 1.50	3.46 ± 0.3
**(B)**			
**AVP1**	**[NaCl] (mM)**		
**Parameter**	**0**	**50**	**100**
V_m_ (U/mg prot.)	0.51 ± 0.01	0.40 ± 0.01	0.24 ± 0.05
L	17 ± 5	50 ± 3	106 ± 14
K_R_ (μM)	84 ± 9	64 ± 13	69 ± 30
**MVP**	**[NaCl] (mM)**		
**Parameter**	**0**	**50**	**100**
V_m_ (U/mg prot.)	0.46 ± 0.01	0.47 ± 0.09	0.34 ± 0.04
L	615 ± 45	123 ± 22	7 10^3^ ± 790
K_R_ (μM)	62 ± 8	90 ± 16	35 ± 11

In order to model the kinetic properties of AVP1 and MVP and relate them to the structural information available for these proteins, a simplified equation was derived from the MWC concerted allosteric model (Monod et al., 1965). The following assumptions were made to obtain the equation:

1. The ratio v/V_m_ is proportional to the saturation function (Ŷ) of the allosteric model
2. AVP1 and MVP can exist in two states, R and T, in thermodynamic equilibrium driven by the allosteric constant L= [T]/[R]. R is assumed to be active and T inactive.
3. The substrate (Mg_2_PPi) binds only to the R state, thus defining a single dissociation constant K_R_.
4. The number of catalytic binding sites for substrate is 2 for both mPPases functional oligomers. This assumption is based on the structural information currently available, which shows that AVP1 and MVP are homodimers with one substrate binding site in each subunit (Li et al., 2016).
5. Mg_2_PPi may also act as an allosteric positive effector by displacing the R-T equilibrium to the R state. To account for this effect in the simplest possible way, L is divided by the factor (1+S/K_R_)^2^. This means that either Mg_2_PPi exerts its stabilising role by binding to the two existing catalytic sites (that is, there are no specific regulatory sites for Mg_2_PPi), or it binds with the same affinity to regulatory and catalytic sites.

The final equation obtained was:

(Equation 1) $${\text{v/V}}_{\text{m}}=\frac{\left(\text{S}/{\text{K}}_{\text{R}})\cdot (1+\text{S}/{\text{K}}_{\text{R}}\right)}{{\left(1+\text{S}/{\text{K}}_{\text{R}}\right)}^{2}+[\text{L}/{\left(1+\text{S}/{\text{K}}_{\text{R}}\right)}^{2}]}$$

Where S is the concentration of the substrate (Mg_2_PPi), K_R_ is the dissociation constant of the substrate toward the active R state, and L is the allosteric constant ([T]/[R]).

The experimental data were fitted with this equation (**Figure 4**), giving plots very similar to those obtained with the empirical Hill equation (data not shown).

The values of the parameters in Equation 1 calculated using RStudio illustrated the differences observed in the kinetic properties of AVP1 and MVP, thus, the allosteric constant L was one order of magnitude higher for MVP than for AVP1 (**Table 3**). This result is consistent with the higher values of the Hill parameter *n_H_* previously obtained (see above). The value of L for AVP1 increased with the concentration of NaCl, whereas the calculated dissociation constant K_R_ did not change significantly (**Table 3B**). This suggests that NaCl displaces the allosteric equilibrium of AVP1 toward the inactive T state. By contrast, in the case of MVP, L decreases in the presence of 50 mM NaCl, while a concentration of 100 mM is necessary to increase its value one order of magnitude with respect to that obtained in the absence of salt (**Table 3B**).

**Figure 5:**
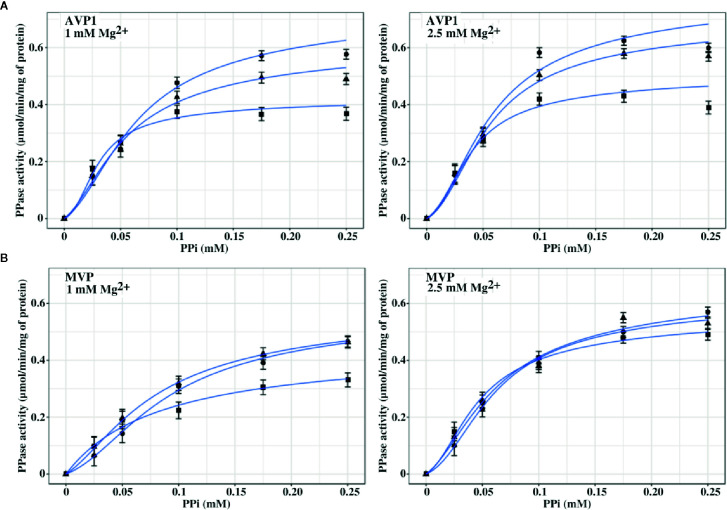
Graphical representation of PPase activity versus PPi concentration for AVP1 **(A)** and MVP **(B)** in the presence of 0 mM (●), 50 mM (▲), and 100 mM (■) NaCl. MgCl_2_ was fixed in the assays at the indicated concentrations. As in **Figure 4**, basal concentrations of Na^+^ four times higher than those of PPi are present in the assays. Data points correspond to the averages ± SE of 3 independent experiments. Graph lines were calculated with Equation 1 using RStudio, as described in Materials and Methods.

In another set of experiments, PPase activities were assayed by fixing MgCl_2_ concentrations in the assays (1 and 2.5 mM) and increasing PPi (up to 0.25 mM) in the presence of 0, 50, and 100 mM NaCl. The concentrations of PPi and Mg^2+^ were chosen to ensure that a significant amount of free Mg^2+^ (that is, not complexed by PPi) was always present in the assay. **Figure 5** shows that the kinetic behaviour of both AVP1 and MVP changed significantly in this situation with respect to the results shown in **Figure 4**. Fitting these data with the Hill equation produced good plots and the V_m_ values were correctly predicted but the values of the rest of the parameters (*n_H_* and K_0.5_) showed high values of standard errors and were not considered reliable (not shown).

Equation 1 predicted the kinetic behaviour of AVP1 and MVP in the presence of excess Mg^2+^ and the values obtained for the different parameters are shown in **Table 4**. The values of the allosteric constant L significantly decreased with respect to those shown in **Table 3**, especially in the case of MVP; moreover, these values were not affected by the presence of increasing concentrations of NaCl, with the only exception of AVP1 when its PPase activity was assayed at 100 mM NaCl and 1 mM MgCl_2_.

**Table 4:** Values of parameters obtained by fitting Equation 1 to kinetic data corresponding to AVP1 (A) and MVP (B) in the presence of fixed Mg^2+^ concentrations.

**(A)**			
**[MgCl_2_] = 1 mM**	**[NaCl] (mM)**		
**Parameter**	**0**	**50**	**100**
**V_m_ (U/mg prot.)**	0.77 ± 0.11	0.64 ± 0.05	0.43 ± 0.05
**L**	4 ± 2	3 ± 1	13 ± 5
**K_R_ (µM)**	56 ± 8	48 ± 2	20 ± 3
**[MgCl_2_] = 2.5 mM**	**[NaCl] (mM)**		
**Parameter**	**0**	**50**	**100**
**V_m_ (U/mg prot.)**	0.82 ± 0.09	0.73 ± 0.05	0.52 ± 0.04
**L**	5 ± 1	7 ± 2	9 ± 3
**K_R_ (µM)**	50 ± 3	42 ± 4	27 ± 4
**(B)**			
**[MgCl_2_] = 1 mM**	**[NaCl] (mM)**		
**Parameter**	**0**	**50**	**100**
V_m_ (U/mg prot.)	0.62 ± 0.05	0.62 ± 0.02	0.45 ± 0.07
L	4 ± 3	2 ± 1	0 ± 1
K_R_ (µM)	83 ± 28	77 ± 6	88 ± 47
**[MgCl_2_] = 2.5 mM**	**[NaCl] (mM)**		
**Parameter**	**0**	**50**	**100**
V_m_ (U/mg prot.)	1.07 ± 0.45	0.69 ± 0.11	0.58 ± 0.03
L	0 ± 1	3 ± 1	5 ± 2
K_R_ (µM)	200 ± 50	59 ± 38	40 ± 4

Values of specific PPase hydrolytic activity obtained with PPi concentrations up to 0.175 mM (AVP1) and 0.25 mM (MVP) were used for curve fitting and calculation of parameters. Graphical representations of experimental data and lines calculated with Equation 1 are shown in **Figure 5**.

Kinetic data further show that V_m_ values for AVP1 steadily decreased with NaCl concentration, especially in the presence of 1 mM Mg^2+^, this effect being somewhat attenuated at 2.5 mM Mg^2+^. PPi concentrations of 0.25 mM and higher exerted a significant inhibitory effect on AVP1 activity at the concentrations of MgCl_2_ tested. Equation 1 could not model this effect, therefore, only data obtained with PPi concentrations up to 0.2 mM were used for curve fitting. Vm values for MVP only decreased significantly at 100 mM NaCl and 1 mM Mg^2+^. Inhibition of MVP by PPi concentrations up to 0.5 mM was not observed (not shown).

**Figure 6:**
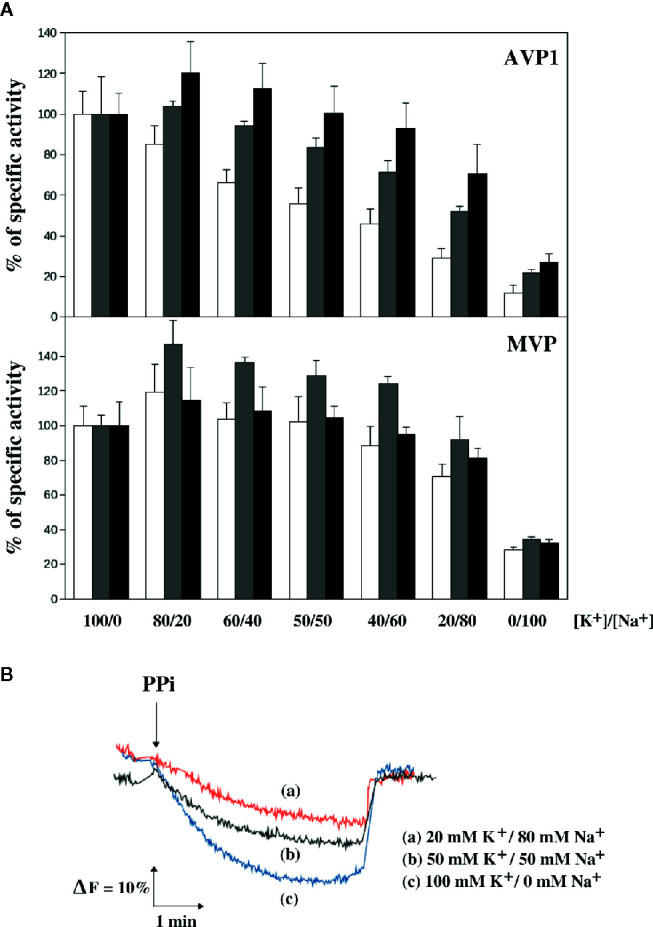
Sensitivity of the PPase activity of AVP1 and MVP **(A)** and the H^+^-pumping activity of AVP1 **(B)** to the [K^+^]/[Na^+^] ration in the presence of different concentrations of Mg^2+^. PPase assays were performed with 0.25 mM PPi and 0.5 mM (white bars), 1 mM (gray bars), and 2.5 mM (black bars) MgCl_2_. Activities are averages ± SE of 4 independent experiments and are expressed as percentages of PPase activity with respect to the average values obtained in the absence of NaCl (a tetrasodium salt of PPi was used, therefore, an extra 1 mM Na^+^ was always present in the assays). These values, expressed as micromoles of PPi/min/mg of protein, were: 0.49 ± 0.05 (0.5 mM Mg^2+^), 0.60 ± 0.11 (1 mM Mg^2+^), 0.62 ± 0.06 (2.5 mM Mg^2+^) for AVP1; and 0.49 ± 0.06 (0.5 mM Mg^2+^), 0.50 ± 0.05 (1 mM Mg^2+^), 0.60 ± 0.08 (2.5 mM Mg^2+^) for MVP. H^+^-pumping activity was assayed as described in Materials and Methods in the presence of 1 mM MgCl_2_ at the indicated concentrations of NaCl.

An appropriate [K^+^]/[Na^+^] ratio in the cytosol has been reported to be extremely important for cell viability in both yeast and higher plants and the presence of high external sodium concentrations may alter this ratio producing toxic effects (Gómez et al., 1996; Maathuis and Amtmann, 1999). PPase activity assays of AVP1 and MVP performed with optimal substrate concentration and decreasing [K^+^]/[Na^+^] ratios showed that AVP1 is inhibited to a higher extent than MVP when the concentration of Na^+^ is higher than that of K^+^. As observed in previous experiments, the presence of excess Mg^2+^ significantly protected AVP1 against inhibition by Na^+^ (**Figure 6A**). Expectedly, the H^+^-pumping activity of AVP1 decreased in parallel with the PPase activity (**Figure 6B**), whereas no H^+^-translocation was detected with microsomes obtained from cells expressing MVP (not shown).

### Intracellular PPi Levels

**Figure 7:**
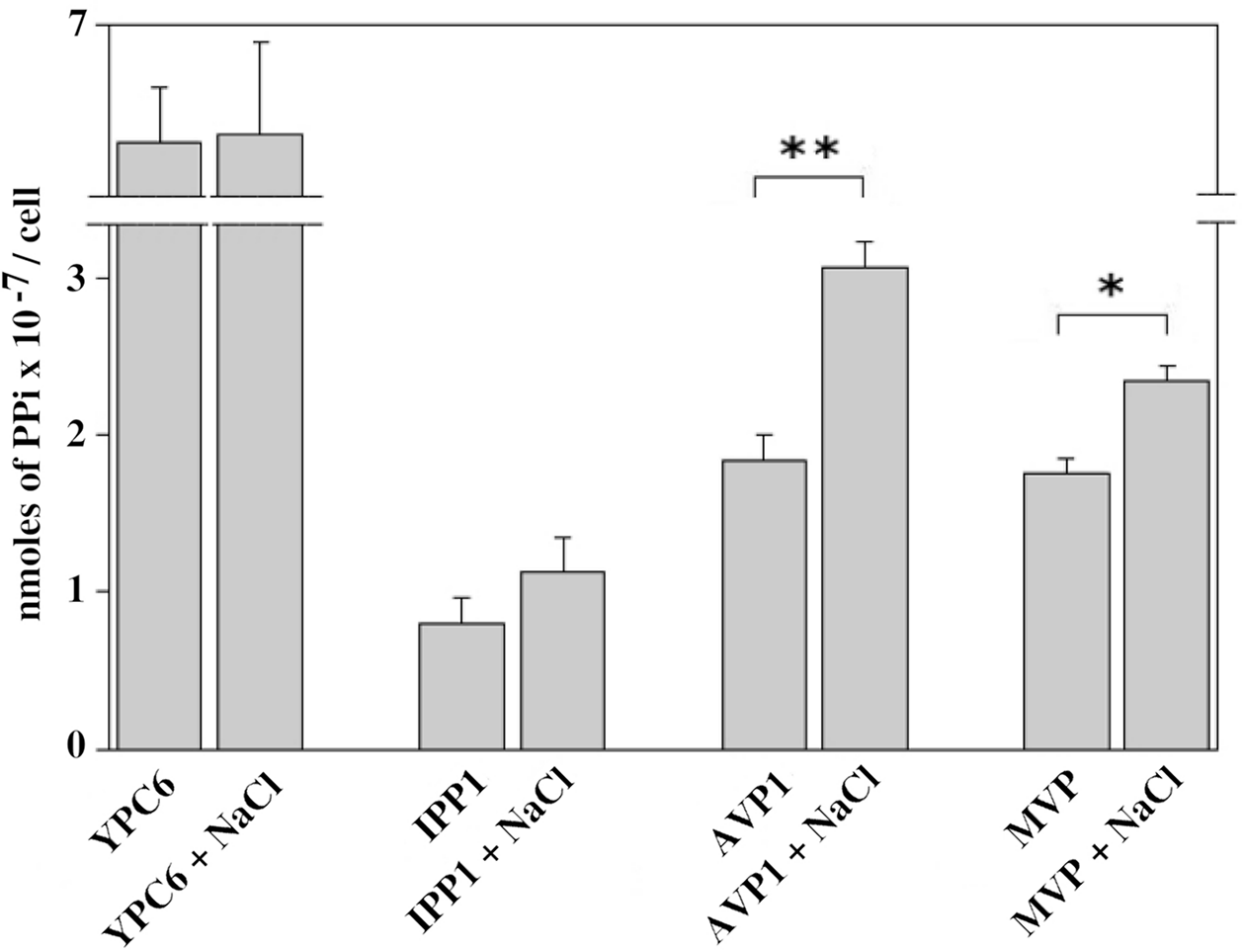
Internal levels of PPi measured in YPC6 cells transformed with the indicated plasmids and grown in the presence of 100 mM NaCl. Experiments were performed as described in Materials and Methods. Values are averages ± SE corresponding to 5 independent experiments. Student’s unpaired *t* tests were done by using webpage http://www.graphpad.com/quickcalcs/ttest1.cfm: (**) Extremely statistically significant difference (P = 0.0006). (*) Very statistically significant difference (P = 0.0026).

In order to check whether the sensitivities of AVP1 and MVP to NaCl *in vitro* could have an effect *in vivo*, YPC6 cells transformed with plasmids pInvGFPAVP1 and pInvGFPMVP were subjected to salt stress with 100 mM NaCl for several hours and cellular levels of PPi were determined. Yeast cells transformed with pInvGFPAVP1 showed significantly higher levels of PPi with respect to control cells expressing IPP1, this effect being milder in those cells transformed with pInvGFPMVP. PPi levels were virtually unchanged in YPC6 cells transformed with plasmid pIPP1 when they were subjected to salt stress (**Figure 7**).

### Addition of Extracellular Magnesium

*In vitro* kinetic studies suggested that magnesium cations can protect mPPases against inhibition by Na^+^ (see above). In order to study a possible protective effect exerted by Mg^2+^ *in vivo*, different concentrations of MgCl_2_ were added to agar plates made with YPD at pH 6, and growth of YPC6 mutants transformed with plasmids pIPP1, pInvGFPAVP1, and pInvGFPMVP was checked in the presence of 100 mM NaCl. There was an improvement of growth in all cases when MgCl_2_ concentration in the medium was raised up to 25 mM (**Supplementary Figure S2**). This suggests a general enhancement of yeast metabolism under salt stress regardless of the enzyme responsible for the removal of cytosolic PPi. This protective effect was not observed at 10 mM MgCl_2_ (not shown).

## Discussion

### Yeast Strains With Conditional Expression of IPP1 Allow Comparative Studies Between Different Heterologously-Expressed PPases

In a paper published in 1999, Gaxiola and co-workers reported that overexpression of AVP1, the major H^+^-PPase from *A. thaliana*, alleviated the sensitivity of yeast toward NaCl. The authors proposed that AVP1 acted by enhancing Na^+^ transport from the cytosol into the vacuole (Gaxiola et al., 1999). These experiments were carried out with a yeast mutant devoid of the P-type Na^+^-ATPases located at the plasma membrane, encoded by the *ENA* genes. In *S. cerevisiae*, these genes are located in chromosome IV in tandem repeats with a variable number of copies (four in the case of strain W303-1A/B, used in that study). Disruption of *ENA* genes results in mutant cells (*ena1-4Δ*) with a higher sensitivity toward Na^+^ than the parental strain (Quintero et al., 1996). This mutant strain was transformed with a plasmid bearing the coding sequence of AVP1 under the control of the *GAL1* promoter and the effect of salt on the transformants was studied. Several aspects of this experimental system are worth considering: first, a version of AVP1 with a point mutation that increased its capacity to pump protons (Zhen et al., 1997) was used. This was probably needed in order to observe phenotypes in yeast, due to the presence of a highly efficient cytosolic sPPase (IPP1) that competes with AVP1 for their common substrate, inorganic pyrophosphate (PPi). Second, the sensitivity to NaCl was checked on galactose because expression of AVP1 would be repressed by glucose, because of the *GAL1* promoter. The use of galactose for studies of Na^+^ toxicity in *S. cerevisiae* must be taken with caution because osmotic adjustment through glycerol synthesis has been reported to be the limiting factor for growth on this carbon source under salt stress (Rios et al., 1997).

In this work, a different experimental approach has been followed. We have previously shown that the glucose-repressible *GAL1* promoter can be inserted immediately upstream the coding sequence of *IPP1* gene in the yeast haploid strain W303-1A. This manipulation generates a strain, YPC3, that is unable to grow on glucose due to the negligible levels of cytosolic sPPase (IPP1) in the presence of this carbon source. This demonstrates the essentiality of cytosolic PPi removal for cell viability (Drake et al., 2010; Serrano-Bueno et al., 2013). Here, the same strategy was applied to an *ena1-4Δ* mutant (G19) and a double *ena1-4Δ nha1Δ* mutant (B31) in order to generate strains YPC5 and YPC6, respectively. Mutant B31 lacks not only *ENA1-4*, but also *NHA1*, that encodes a K^+^(Na^+^)/H^+^ antiporter located at the plasma membrane; expectedly, this strain exhibits a higher sensitivity to sodium than the *ena1-4Δ* mutant G19 (Bañuelos et al., 1998). The derived strains YPC5 and YPC6 maintained their sensitivities toward Na^+^ but they could not grow on glucose due to the repression of *IPP1* transcription (**Figure 2**).

Transformation of YPC3, YPC5, and YPC6 with plasmids bearing genes coding for mPPases under the control of a constitutive promoter restored growth on glucose, as expected from previous reports (Pérez-Castiñeira et al., 2002; Drake et al., 2010; Pérez-Castiñeira et al., 2011; Hernández et al., 2015; Hernández et al., 2016). This experimental approach has several advantages: (a) it reproduces in yeast a situation similar to that of the plant cell, where most of the PPi generated in the cytosol is removed by AVP1 (Ferjani et al., 2011; Kriegel et al., 2015; Segami et al., 2018b); (b) the use of yeast strains that depend on the expression of fully functional mPPases for growth allows comparative studies on how salt stress may affect these proteins *in vivo* in the same cellular environment; (c) the use of glucose, the preferred carbon source of yeast, ensures that the inhibitory effects exerted by NaCl on growth are mainly due to sodium toxicity rather than to osmotic stress (Rios et al., 1997).

### The PPase Activity of AVP1 Is More Sensitive to Na^+^ Than That of MVP

Our results show that yeast cells expressing AVP1 always grew worse in the presence of NaCl than those expressing MVP or the yeast cytosolic sPPase (IPP1). Moreover, the fact that none of the mPPases tested was able to support growth on NaCl better than IPP1 hinted that the former were not able to reduce significantly the concentration of Na^+^ in the cytosol. Moreover, the chimera TcGFPAVP1, that is predominantly located in internal yeast membranes and complements the vacuolar H^+^-ATPase (Pérez-Castiñeira et al., 2011; Hernández et al., 2015; Hernández et al., 2016), supports growth under salt stress less efficiently than InvGFPAVP1, that preferentially locates at the plasma membrane (Drake et al., 2010). This suggests that, in our system, AVP1 is not increasing the proton gradient across the vacuole membrane, that would result in an enhancement of secondary Na^+^ transport into this compartment. Altogether, these results indicate that the observed phenotypes depend on the PPase activities rather than on the ion pumping capacity of the mPPases.

Total intracellular concentrations of Na^+^ and K^+^ were measured, as previously reported (Gaxiola et al., 1999), showing that the [K^+^]/[Na^+^] ratio significantly decreased when transformed YPC6 cells were grown in the presence of 100 mM NaCl regardless of the PPase (soluble or membrane-bound) they expressed. Moreover, analyses of Na^+^ and K^+^ concentrations in the cytosol, performed by selectively making the plasma membrane permeable (Ohsumi et al., 1988), yielded similar results (not shown). These results discarded a significant extrusion of Na^+^ ions from the cytosol mediated either by AVP1 [coupled to H^+^/Na^+^ antiporters (Nass et al., 1997)] or by MVP (directly).

Other experiments aimed at establishing possible mechanisms of AVP1 inhibition by Na^+^ *in vivo* were performed with negative results. These included studies of trypsin digestion patterns, to check for conformational changes produced by salt stress in AVP1, or immunoprecipitation, to look for possible interactions between AVP1 and proteins involved in stress response (data not shown). Finally, the direct effect of Na^+^ on the two activities presented by AVP1 (PPi hydrolysis and proton pumping) was tested. The possible effects on the Na^+^ sensitivity of AVP1 and MVP exerted by Mg^2+^ was also studied, as previous evidence points out the importance of this divalent cation not only for the kinetic and catalytic properties but also for the structures of all types of PPases (Heikinheimo et al., 1996; Kellosalo et al., 2012; Lin et al., 2012; Tsai et al., 2014).

**Figure 4** shows that AVP1 is more sensitive to NaCl than MVP when the concentration of Mg^2+^ exactly doubles that of PPi. AVP1 and MVP showed different degrees of cooperativity, the deviation of the latter from the Michaelian behaviour being more pronounced than that of AVP1. This was illustrated by the higher values of *n_H_* and the allosteric constant L obtained for MVP when the experimental data were fitted with the Hill equation and an expression derived from the concerted allosteric model, respectively. Moreover, the degree of cooperativity (and hence the value of L) of AVP1 increased with NaCl concentration, whereas, in the case of MVP, this effect was significant only at high concentrations of salt (100 mM). Therefore, according to our simplified allosteric model, Na^+^ is displacing the equilibrium between the active R state and the inactive L state toward the latter, that is, it acts as a negative allosteric effector, mainly for AVP1.

The presence of free Mg^2+^ significantly decreases the value of the predicted allosteric constant, which suggests that this cation stabilizes the active R state of both AVP1 and MVP. Addition of 100 mM NaCl in the assays under these conditions results in a more modest increase of L with respect to previous experiments, that were performed with no excess of Mg^2+^.

In summary, a simplified version of the allosteric model supports the idea that mPPases have at least two conformational states with different catalytic activities, with Mg^2+^ acting both as a co-substrate and as a positive allosteric effector; by contrast, Na^+^ is a negative allosteric effector, especially for AVP1. These results are consistent with previous reports published by other groups for this type of proteins (Takeshige and Hager, 1988; Luo et al., 1999; Hsiao et al., 2004). Interestingly, exhaustive kinetic studies done with the Na^+^-regulated H^+^-PPase from the green sulphur bacteria *Chlorobium limicola* expressed in *E. coli* indicate the Na^+^ can displace Mg^2+^ from the enzyme, thereby arresting substrate conversion (Luoto et al., 2015).

### Limitations of the Kinetic Studies

Our results differ from those previously reported by Malinen et al. (2008), that thoroughly studied the kinetic behaviour of MVP and found that the dependence of the hydrolysis rate on substrate concentration obeyed the Michaelis-Menten equation. In this study, MVP was heterologously expressed in *E. coli*, that may imply significant changes in the protein (folding, interaction with biomembranes, and/or post-translational modifications, among others) with respect to our approach, based on the expression of chimeric versions of mPPases in yeast. Moreover, the use of different PPase activity assays (Rathbun and Betlach, 1969; Baykov and Avaeva, 1981) might also contribute to the differences observed by the two groups in the kinetic properties of MVP.

We are aware of the limitations of our experimental approach to study kinetic properties and that many more data, as well as a more elaborated model, are needed in order to establish with precision the mechanistic differences between AVP1 and MVP. In any case, our aim was not to perform exhaustive kinetic studies of AVP1 and MVP, but to compare the properties of both mPPases in the same cellular environment in order to find a correlation between the observed phenotypes and the enzymatic activities measured *in vitro*. The evidence presented here shows that the PPase activity of MVP is less sensitive to salt stress than AVP1 when both proteins are embedded in membranes of a eukaryotic organism such as yeast. This is further supported by the results shown in **Figure 6**, that indicate that low free Mg^2+^ concentrations and [K^+^]/[Na^+^] ratios significantly inhibit both the hydrolytic and the proton pumping activities of AVP1 at optimal PPi concentration. Moreover, intracellular PPi levels *in vivo* are significantly increased in yeast YPC6 cells expressing InvGFPAVP1 grown in the presence of 100 mM NaCl with respect to cells grown in culture medium without added salt. The increase was lower in cells expressing InvGFPMVP, which might explain why these cells are more resistant to salt stress, since PPi accumulation collapses anabolism (Lahti, 1983; Serrano-Bueno et al., 2013).

### A Possible Role of Mg^2+^ in Salt Tolerance

Addition of external magnesium in the culture medium alleviated the sensitivity to NaCl in all the transformants obtained using the YPC6 mutant, even in cells expressing IPP1 (**Supplementary Figure S2**). This suggests that Mg^2+^ exerts a general beneficial effect under conditions of salt stress, which is not surprising, as Na^+^ has been reported to inhibit other enzymes, such as Hal2 nucleotidase and the RNase MRP, by displacement of Mg^2+^ from its binding sites (Serrano et al., 1999).

Homeostasis of Mg^2+^ in yeast is mechanistically complex and several transporters and internal reservoirs seem to be implicated (Klompmaker et al., 2017), moreover, this cation has been reported to influence the K^+^/Na^+^ exchange rate in this organism (Rodríguez-Navarro and Sancho, 1979). This evidence suggests that introducing Mg^2+^ to study mechanisms of salt stress complicates the overall picture and deserves more experimental work. In any case, it might be interesting to study if the toxic effects exerted by Na^+^ in yeast and other organisms might be linked to depletion of free Mg^2+^ levels in the cytosol.

### Controversy About the Mechanisms by Which H^+^-PPases Alleviate Salt Stress

The results presented here suggest that Na^+^ can directly inhibit AVP1 *in vivo*, which can explain why an overexpression of this protein increases salt tolerance in plants. AVP1 inhibition would result in an accumulation of PPi, that might alter the reactions catalysed by the PPi-dependent phosphofructokinase and the UDP-glucose pyrophosphorylase, along with other anabolic reactions (Ferjani et al., 2018). Moreover, higher PPi concentrations could also decrease the concentration of free divalent cations such as Mg^2+^ and Ca^2+^ by chelation, thereby altering the activity of multiple proteins and cellular systems (Segami et al., 2018a).

There is a certain controversy about the mechanism by which overexpression of AVP1 and its ortologs increases tolerance to abiotic stresses in *A. thaliana* and other crops. It is often considered that H^+^-PPase overexpression increases the pH gradient across the tonoplast thus promoting the removal of Na^+^ from the cytosol *via* proton-coupled Na^+^-K^+^ transporters. This view was reinforced by a recent report showing that transient overexpression of the H^+^-PPase in *Nicotiana benthamiana* leads to higher vacuolar proton currents and vacuolar acidification (Graus et al., 2018). This results in a drop in photosynthetic capacity, plasma membrane depolarization and eventual leaf necrosis under non-stressed conditions; paradoxically, salt rescued leaf cells from cell death. Moreover, in non-transformed plants, a rise in V-PPase but not of V-ATPase pump currents was detected in the presence of salt. These results indicate, on the one hand, that plants need to regulate the H^+^-PPase pump activity very carefully and, on the other, that H^+^-PPase proton pump function becomes increasingly important under salt stress (Graus et al., 2018). However, there are some points to be considered in this communication, thus, a 300% higher H^+^-PPase proton pump activity with respect to control plants was obtained by transient overexpression. This is much higher than the values obtained by stable overexpression of H^+^-PPases in other plants and definitely becomes detrimental under non-stressed conditions. The latter is probably due to the impairment of cellular pH homeostasis, therefore, salt-stress is likely to alleviate this phenotype by readjusting cellular pH *via* H^+^/Na^+^-K^+^ antiporters. Another interesting result that appears in this article is that *N. benthamiana* plants overexpressing IPP1 showed no detrimental effects in the absence of salt but presented similar maximum photochemical quantum yields of photosystem II to those overexpressing H^+^-PPases in the presence of 200 mM NaCl (Graus et al., 2018). This shows that very high expressions levels of H^+^-PPases may not give any advantage to the plant compared with the overexpression of a soluble PPase, such as IPP1. In a way, this resembles the results presented here.

Several groups have reported that stable overexpression of H^+^-PPases stimulates growth and increases tolerance to biotic and abiotic stresses of *A. thaliana* and many other plants, however, in these reports plants overexpressing other types of PPases were never used as controls (Gaxiola et al., 2001; Park et al., 2005; Gao et al., 2006; Li et al., 2008; Lv et al., 2008; Pasapula et al., 2011; Schilling et al., 2014; Yang et al., 2015; Lv et al., 2016; Ahire et al., 2018). This makes difficult to ascertain the degree of involvement of the two activities exhibited by H^+^-PPases (PPi hydrolysis and H^+^-pumping) in stress tolerance. On the other hand, it has been reported that salt tolerance in *A. thaliana* is positively correlated with the expression of *AVP1* and *AtNHX1*, the gene coding for a vacuolar Na^+^/H^+^ antiporter, in both roots and shoots. However, no measures of protein levels or enzymatic and ion transport activities were performed in this study, therefore, the physiological implications of these results were not established (Jha et al., 2010).

Our results are consistent with experiments performed in *A. thaliana* that suggest that the hydrolysis of cytosolic PPi, rather than vacuolar acidification, is the major function of AVP1 *in planta* (Ferjani et al., 2011; Kriegel et al., 2015; Segami et al., 2018b). In any case, the cytosolic concentration of PPi may have many implications for metabolism, especially in plant cells (Segami et al., 2018a). This must be taken into consideration when using PPases to develop plants with increased tolerance to different stresses.

### Phylogenetics Analysis

**Figure 8:**
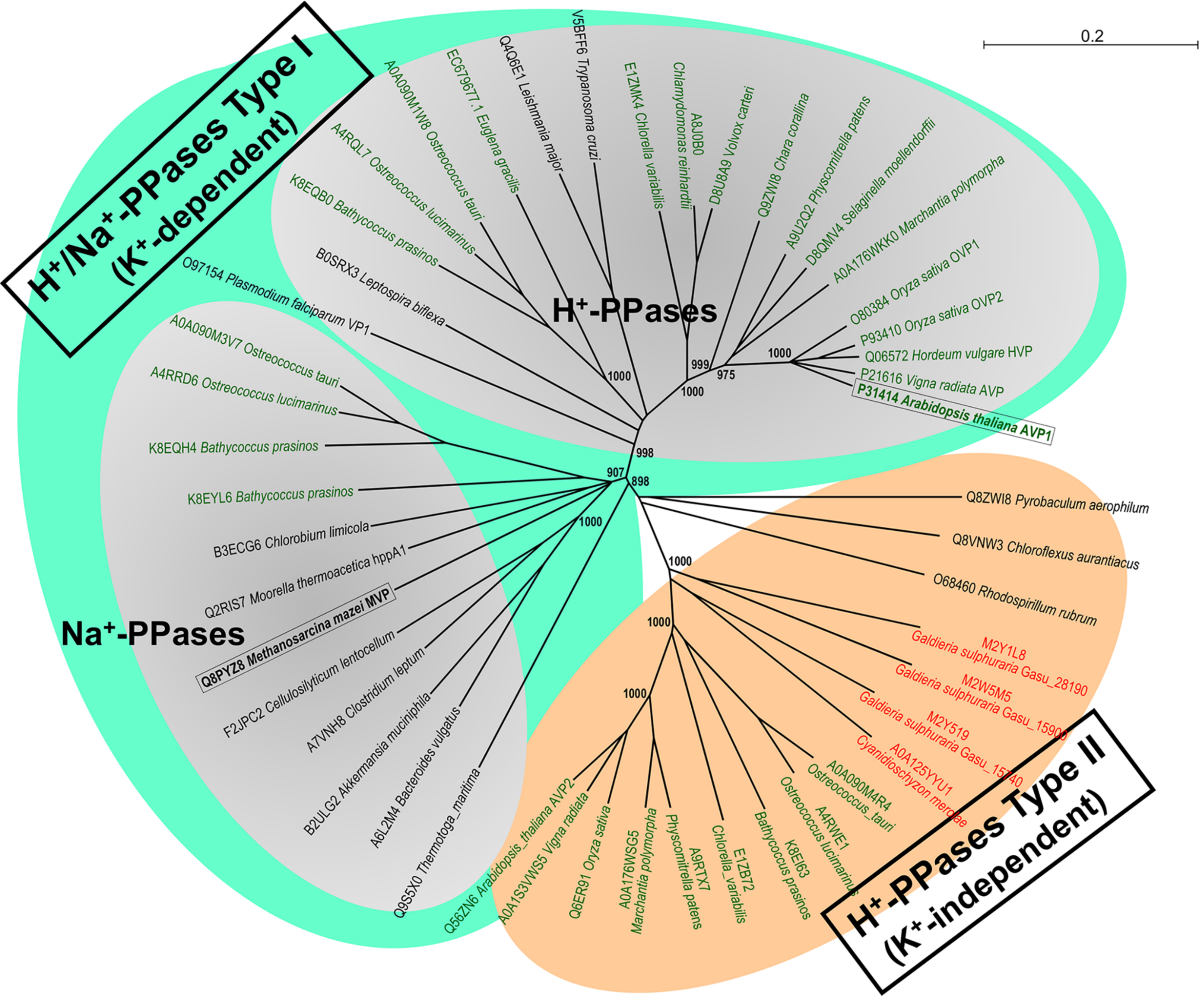
Molecular phylogenetic analysis of the three major classes of mPPases, K^+^-dependent H^+^- and Na^+^-PPases and K^+^-independent H^+^-PPases, broadly distributed among prokaryotes, protists and the photosynthetic lineage (algae and plants). A Neighbor-Joining phylogenetic tree obtained from a multiple sequence alignment of selected protein orthologs from prokaryotes, microalgae, parasitic protists and higher plants (generated by CLUSTAL X and Sea View v5.2 software packages) is shown. Note the well-defined and robust clusters of the three main classes of H^+^- and Na^+^-PPases, which are clearly divergent, and the probable Na^+^-PPase orthologs of marine prasinophycean microalgae (*Ostreococcus* spp.; *Bathycoccus* spp.) that clearly cluster with *bona fide* Na^+^-PPases of bacteria and archaea. A similar topology was obtained for a Maximum Likelihood tree (not shown). The two mPPases studied in this work are shown boxed in bold. Those of land plants and Chlorophyta microalgae are shown in green types and those of Rhodophyta microalgae in red types. Sequences are identified by their species names and UniProtKB accession numbers or genome/transcriptome sequencing projects numbers. The numbers in selected nodes are bootstrap values based on 1,000 replicates. Scale bar indicates number of changes per amino acid site.

Based on molecular phylogenetic analysis and biochemical evidences, mPPases can be classified into three major classes: K^+^-dependent H^+^- and Na^+^-PPases, and K^+^-independent H^+^-PPases (Serrano et al., 2007; Baykov et al., 2013). Phylogenetic analyses suggest that these ancestral ionic pumps are broadly distributed among prokaryotes (bacteria and archaea), protists, and the photosynthetic lineage (algae and plants), being more widespread than previously thought (**Figure 8**). The mPPases studied in this work, AVP1, from a land plant, and MVP, from a salt-tolerant archaeon, are well characterized archetypical representatives of their respective mPPase classes and are evolutionarily rather distant (**Figure 8**), which would be consistent with their different sensitivities to sodium. Although the physiological significance of Na^+^-PPases and their involvement in salt tolerance are still to be clarified, the higher tolerance of MVP to sodium inhibition reported in this work strongly support a role in such scenario. Since molecular phylogenetic studies carried out by our group (A. Serrano, in preparation) indicate that orthologs of these prokaryotic Na^+^-PPases appear in several groups of marine salt-tolerant photosynthetic protists (e.g., Prasinophytes) (**Figure 8**), the generation of transmembrane Na^+^-gradients at the expense of PPi could be a mechanism of energy transduction common to prokaryotic and eukaryotic microorganisms. The biochemical and functional validation of these eukaryotic Na^+^-PPases, as well as their physiological significance and evolutionary origin, are currently under study by our group.

## Conclusions

Our results not only point at Na^+^-PPases as potential powerful biotechnological tools to obtain salt-resistant plants, but also suggest that altering the subcellular distribution of heterologously-expressed membrane-bound proteins, like mPPases, may also be crucial to obtain the desired phenotypes.

## Data Availability Statement

All datasets generated for this study are included in the article/**Supplementary Material**.

## Author Contributions

Both authors jointly designed the experimental approach. JP-C performed laboratory work and wrote the first version of manuscript. AS performed in silico phylogenetic analyses and corrected and supervised the manuscript.

## Funding

Regional Andalusian Government (Junta de Andalucía) and Spanish Ministry of Science and Innovation for financial support to PAIDI group BIO-261 (grants P07-CVI-03082 and BFU2010-15622 to AS), partially funded by the EU-FEDER program (Fondo Europeo de Desarrollo Regional). PAIDI group BIO-261 belongs to the CeiA3 and AndaluciaTECH University Campuses of International Excellence.

## Conflict of Interest

The authors declare that the research was conducted in the absence of any commercial or financial relationships that could be construed as a potential conflict of interest.

## Abbreviations

AVP1, K^+^-dependent H^+^-translocating inorganic pyrophosphatase from *Arabidopsis thaliana*; mPPase, membrane-bound integral ion (H^+^- or Na^+^) -pumping inorganic pyrophosphatase; MVP, K^+^-dependent Na^+^-translocating inorganic pyrophosphatase from *Methanosarcina mazei;* PPase, Inorganic pyrophosphatase; PPi, Inorganic pyrophosphate; sPPase, Soluble inorganic pyrophosphatase.
