# Supplementary figures and images for: The H+-Translocating Inorganic Pyrophosphatase From Arabidopsis thaliana Is More Sensitive to Sodium Than Its Na+-Translocating Counterpart From Methanosarcina mazei
Source: Front Plant Sci. 2020 Aug 12;11:1240. doi: 10.3389/fpls.2020.01240 (PMC7438732; doi:10.3389/fpls.2020.01240)

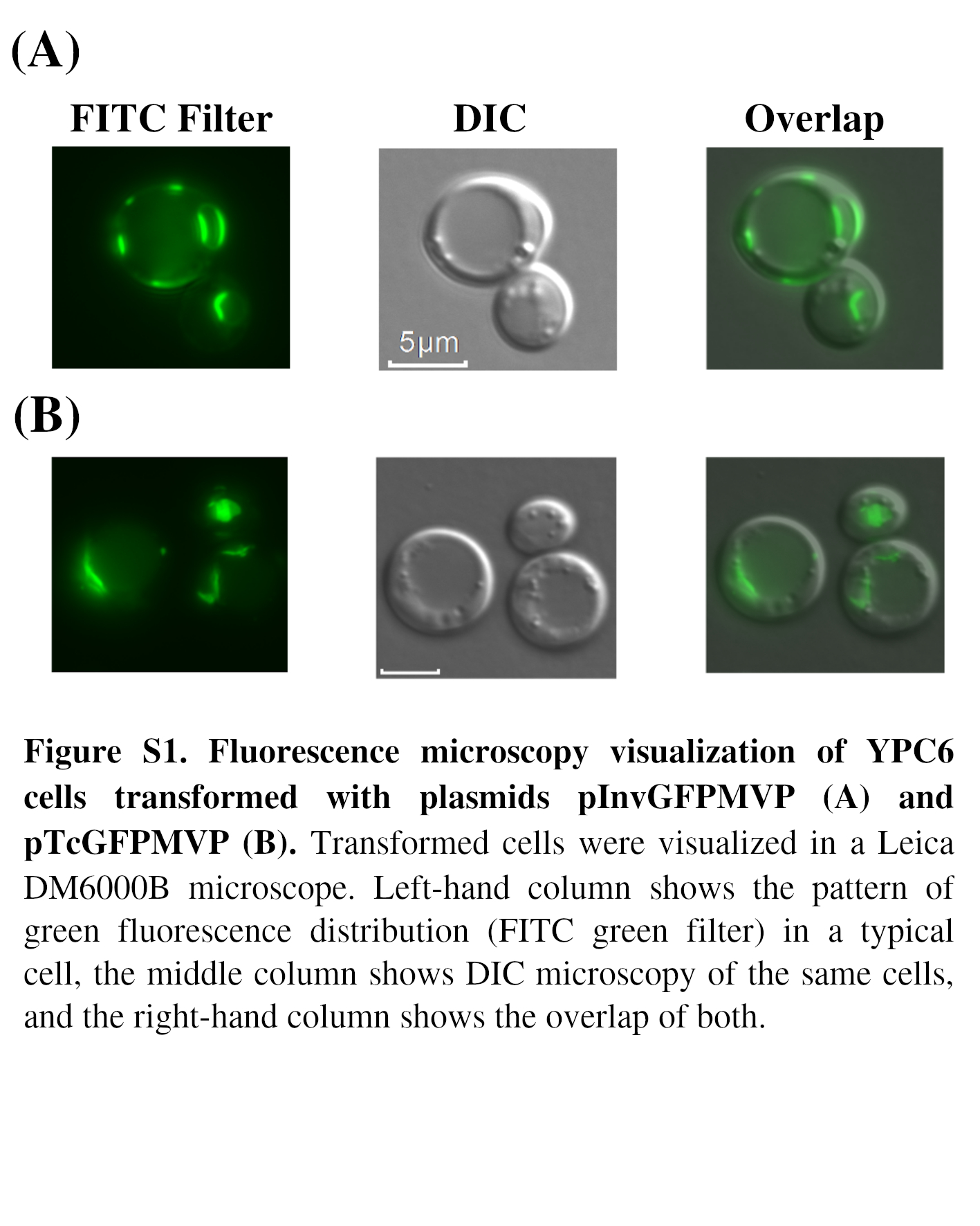

Supplement: Supplementary file 1 [file Image_1.jpeg]

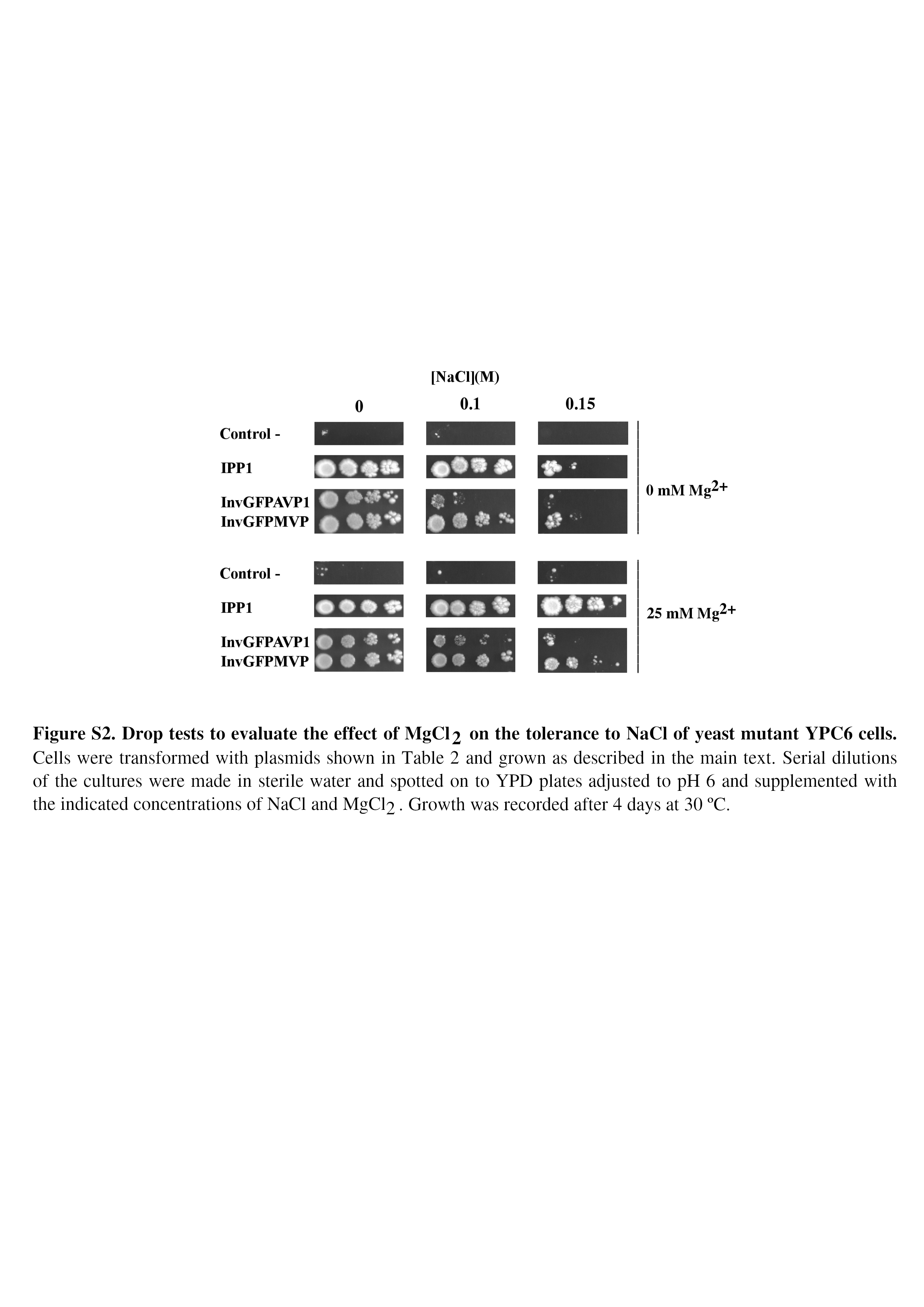

Supplement: Supplementary file 2 [file Image_2.jpeg]
